# A retrospective analysis of the pharmacovigilance data registry in a tertiary teaching hospital in Jordan

**DOI:** 10.1080/20523211.2024.2378461

**Published:** 2024-07-23

**Authors:** Khawla Abu Hammour, Faris El-Dahiyat, Rund Hyari, Sara Salameh, Qusai Manaseer, Rana Abu Farha, Adnan Abu Hammour, Mohammed Zawiah

**Affiliations:** a Department of Biopharmaceutics and Clinical Pharmacy, Faculty of Pharmacy, The University of Jordan, Amman, Jordan; b College of Pharmacy, Al Ain University, Abu Dhabi, UAE; c Pharmacy Department, Jordan University Hospital, Amman, Jordan; d School of Medicine, Orthopedic Department, The University of Jordan, Amman, Jordan; e Department of Clinical Pharmacy and Therapeutics, Faculty of Pharmacy, Applied Science Private University, Amman, Jordan; f School of Medicine, Gastroenterology Department, The University of Jordan, Amman, Jordan; g Department of Clinical Practice, College of Pharmacy, Northern Border University, Rafha, Saudi Arabia; h Department of Pharmacy Practice, College of Clinical Pharmacy, University of Al Hodeida, Al Hodeida, Yemen

**Keywords:** Adverse drug reactions, Pharmacovigilance, Drug safety, ADR reporting patterns, Jordan

## Abstract

**Objectives:**

The study aims to analyse adverse drug reaction (ADR) reporting patterns at Jordan University Hospital to enhance pharmacovigilance practices.

**Methods:**

Retrospective analysis of ADR data from February to August 2023 was conducted. Data included patient demographics, drugs implicated, seriousness criteria, and system organ classes affected.

**Results:**

Among 1340 ADR reports analysed, females accounted for 67.4% of cases, with adults aged 18 to less than 65 years comprising 95.3% of reports. The majority of ADRs were non-serious, with only 2.1% resulting in hospitalisation or prolonged hospital stay. The most frequently reported ADRs included abdominal pain (8.3%), nausea (6.9%), headache (4.7%), and dizziness (4.7%). Notably, cardiovascular system drugs (16.4%) and alimentary tract and metabolism drugs (16.2%) were commonly associated with ADRs, followed by musculoskeletal system drugs (9.0%). Additionally, among all reported drugs, 99.9% were considered suspects, (suspected ADR cases include patient treatment cases for which a likelihood of being related to a drug therapy was scored as ‘possible’, ‘probable’, or ‘certain’ after causality assessment (by the WHO-UMC system in 2017), with oral administration being the predominant route (89.5%).

**Conclusion:**

The study highlights a notable increase in ADR reporting during the study period compared to historical data, indicating heightened awareness and understanding among healthcare providers. Enhanced pharmacovigilance practices, particularly involving pharmacists, are essential for detecting and reporting ADRs effectively. Further investigation into factors contributing to prevalent serious ADRs is warranted to improve patient safety and health outcomes.

## 1. Introduction

The advancement of pharmaceuticals in recent decades has brought remarkable advantages to patients. Medications play a vital role in public health by reducing illness and death, eradicating diseases, enhancing quality of life, and extending life expectancy (Buxbaum et al., 2020). However, it's not uncommon to experience adverse drug reactions (ADRs), which can lead to unfavourable outcomes.

The World Health Organization (WHO) defines ADRs as ‘harmful and unintended responses to medication that occur at typical human dosage levels’ (WHO, 2002). The existing literature is filled with studies that highlight the importance of reporting and analysing ADRs and their outcomes. Research has shown that the prevalence of ADRs leading to hospitalisation varies from 1.0% to 16.8% in the United States (Lazarou et al., 1998), and from 0.5% to 12.8% across European countries (Bouvy et al., 2015). In a review focusing on the Western region, the economic implications of preventable ADRs were analysed. The study revealed a prevalence of 37.9% for preventable ADRs (Formica et al., 2018). Additionally, the research highlighted the substantial direct and indirect costs associated with ADRs, as well as the prolongation of hospital stays resulting from these events (Formica et al., 2018). These findings underscore the financial impact and healthcare burden of preventable ADRs, emphasising the need for effective strategies to mitigate and prevent them. By addressing and minimising preventable ADRs, we can not only improve patient outcomes but also reduce healthcare costs and optimise resource allocation (Awwad et al., 2024; Tabaza et al, 2023).

Pharmacovigilance is the scientific field dedicated to collecting, monitoring, evaluating, and reporting ADRs in order to improve patient safety and health outcomes, additionally, an adverse event is considered serious if it meets one or more of the following criteria: results in death. is life-threatening, requires inpatient hospitalisation or prolongation of existing hospitalisation, results in persistent or significant disability or incapacity, or results in a congenital anomaly (birth defect) (Beninger, 2018). It plays a critical role in the regulatory process for ensuring drug safety. Monitoring and tracking ADRs allow us to identify potential risks and evaluate the effectiveness of medications, and make informed decisions (Beninger, 2018).

The accurate tracking and reporting of ADRs is a key objective for medical institutions and organisations like the WHO. It is crucial to determine the frequency of ADRs occurrences during the post-marketing period, when approved drugs are being used in real-life situations (Alomar et al., 2020). This allows us to gather valuable data on the safety of medications and ensure that any potential risks are identified and addressed promptly. By closely monitoring ADRs, we can enhance patient care and continuously improve the overall safety and effectiveness of drugs on the market.

In Jordan, there are laws and regulations in place to detect and monitor ADRs. A national pharmacovigilance center, which is connected to the Jordan Food and Drug Administration (JFDA), was established and became a full member of WHO's international drug monitoring program in 2002 (WHO, 2022). This national center plays a crucial role in coordinating pharmacovigilance activities across the country, enhancing the knowledge of healthcare professionals and providers about pharmacovigilance, and continuously monitoring medication safety (El-Dahiyat, Abu Hammour, et al., 2023; El-Dahiyat, Hammour, et al., 2023).

The analysis of national pharmacovigilance databases worldwide has uncovered variations in patterns, characteristics, and outcomes of ADRs. These differences can be attributed to factors such as the type of reports analysed (e.g. serious reports), the patient population, specific medications, and drug classes, among others. For instance, in the Nigerian VigiFlow database, a previous study has indicated that a majority of the reported ADRs were associated with antineoplastic and immunomodulating agents, as well as anti-infective drug classes (Ogar et al., 2019). This highlights the specific medication classes that have been observed to contribute significantly to ADRs in that particular region. It underscores the importance of continuous monitoring and analysis of ADR patterns to ensure the safe and effective use of medications, particularly in the context of different regions and patient populations.

Thus, the primary objective of this study was to analyse and understand the patterns of ADRs reported by Jordan University Hospital between Feb 2023 and Aug 2023. Our aim was to identify the number of ADR reports, the most frequently reported ADRs, determine the medications commonly associated with ADRs, assess the body systems predominantly affected by ADRs, and evaluate the consequences of ADRs.

## 2. Method

### 2.1 Study design and setting

This study adopted a retrospective analysis of pharmacovigilance data on ADRs reported by Jordan University Hospital from February 2023 to August 2023. The reporting of ADRs encompassed Jordan University Hospital and the surrounding middle region of Jordan. the chosen period is due to the fact that before this timeframe, ADR reporting at this hospital was limited. Subsequently, collaborative efforts with the JFDA were implemented to improve the reporting system, including: conducting training sessions for healthcare providers in collaboration with JFDA and WHO on pharmacovigilance (PV), activating the PV center within the hospital, and a QR code to facilitate ADR reporting by healthcare providers and patients (Abu Farha et al., 2018, Abu Hammour et al., 2017). These initiatives led to a significant increase in the number of reports, allowing for a more comprehensive analysis of ADRs. This study focuses on analysing ADRs rather than assessing the impact of the aforementioned actions on enhancing the reporting system.

### 2.2 Data collection and management

#### 2.2.1. Data registry

The Rational Drug Use and Pharmacovigilance Department at the JFDA has developed specific printed or digital forms to facilitate spontaneous and voluntary reporting of adverse drug reactions (ADRs) by patients, healthcare providers, and drug companies. These forms adhere to good pharmacovigilance (PV) practices and include sections covering patient demographics, details of the ADR, its severity and consequences, information on the suspected drug, and details about the reporter. At Jordan University Hospital, electronic reporting forms were implemented by the pharmacovigilance team to streamline the ADR reporting process. Submitted ADR forms underwent initial screening and processing by the team, and data were subsequently entered into VigiFlow, a web-based PV management system. This database served as the repository for ADR information, capturing descriptions, suspected drugs causing ADRs, and outcomes. Reports identified as duplicate, invalid, or incomplete were excluded during the initial screening. Drugs implicated in ADRs were categorised using the Anatomical Therapeutic Chemical (ATC) classification system into 14 major drug classes. Drugs assigned multiple ATC classifications were separately tabulated within each group. ADRs and the System Organ Classes (SOCs) affected were classified using the Medical Dictionary for R Serious adverse drug reactions (ADRs) identified by the reporter on the ADR form are classified into one of the following categories: fatal, life-threatening, requiring hospitalisation or extending hospital stay, or resulting in congenital anomalies, persistent disabilities, or other significant medical conditions.

### 2.3 Statistical analysis

Descriptive statistics, such as frequency, and percentages were calculated to describe the demographics, drug, and ADR characteristics. The incidence of reported ADRs was determined, with a focus on the most common occurrences and their respective systems. Data analyses were performed using STATA version 17 (StataCorp), a widely utilised statistical software package (StataCorp LLC, College Station, TX, USA).

## 3. Results

### 3.1 Adverse drug reactions reports and patient demographics

During the study period of monitoring, a total of 1340 individual ADR reports were analysed. These reports encompassed 648 unique medications. Notably, female patients accounted for a higher proportion of ADR reports (n = 908, 67.7%) than males (n = 432 32.2%). The majority of ADR reports were observed in adult patients aged 18 to less than 65 years (n = 1277, 95.3%). Notably, 0.22% of the reports (n = 3) were related to pregnancy. Moreover, in terms of seriousness criteria, the majority of ADRs did not result in serious consequences, as evidenced by the absence of reports related to death, life-threatening situations, or disabling/incapacitating effects, while only 2.1% (n = 28) of the reports were related to hospitalisation or prolonged hospitalisation (Table 1).

**Table 1.** Characteristics of the included patients and ADR (n = 1340).

Parameter	n (%)
**Gender**	
Male	429 (32.0)
Female	903 (67.4)
Missing data	8 (0.6)
**Age group**	
< 18 years	27 (2.0)
18 to < 65 years	1277 (95.3)
≥ 65 years	22 (1.6)
Missing data	14 (1.0)
**Pregnancy**	3 (0.22)
**Seriousness criteria**	
Caused/prolonged hospitalisation	28 (2.1)
Death	0 (0)
Life threatening	4 (0.3)
Disabling/incapacitating	0 (0)
Congenital anomaly/birth defects	0 (0)
Other serious consequences	0 (0)

### 3.2 Distribution of ADEs by drugs and their respective therapeutic classes

**Figure 1.**
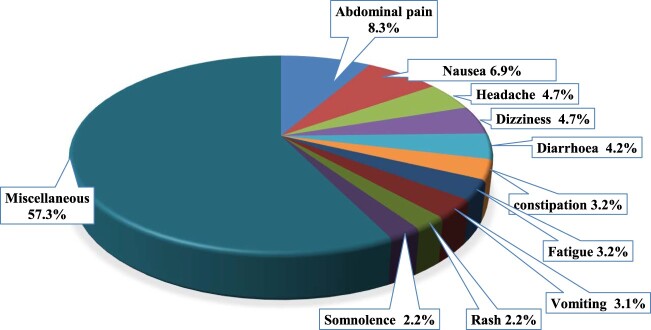
Percentages of the most common reported ADRs (n = 2489).

A total of 2489 ADRs were reported in the analysed 1340 reports (correspond to patients), as depicted in Figure 1. Notably, the top four reported ADRs, including abdominal pain (8.3%), nausea (6.9%), headache (4.7%), and dizziness (4.7%), accounted for approximately one-quarter of these adverse reactions. The following adverse drug reactions (ADRs) were each reported once in the ADR reports: teeth pain, thrombocytopenia, runny nose, and increased saliva.

**Figure 2.**
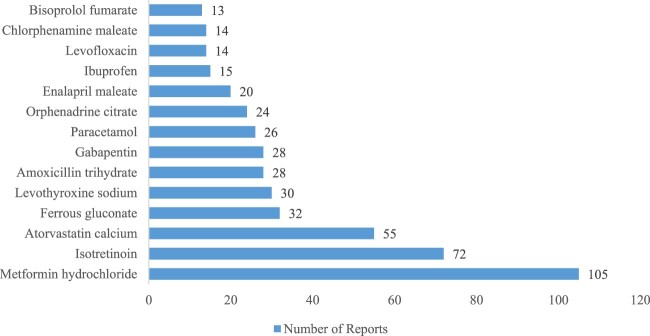
Frequency of the top reported medications in ADR reports.

The principal 15 frequently reported drugs for ADRs are depicted in Figure 2. Metformin hydrochloride (105,7.8%), Isotretinoin (72,5.4%), Atorvastatin calcium (55, 4.1%), Ferrous gluconate (32, 2.4%), Levothyroxine sodium (30, 2.2%), Amoxicillin trihydrate Clavulanate potassium (28, 2.1%), Gabapentin (28, 2.1%), Paracetamol (26, 1.9%), ‘Orphenadrine citrate, Paracetamol’ (24, 1.8%), Enalapril maleate (20, 1.5%), Ibuprofen (15, 1.1%), Levofloxacin (14, 1.0%), Chlorphenamine maleate (14, 1.0%), Bisoprolol fumarate (13, 0.97%) were the most frequent for ADR reports.

The most commonly reported ATC drug classes (Table 2) were cardiovascular system (n = 221, 16.4%), followed closely by alimentary tract and metabolism (n = 218, 16.2%). Additionally, musculoskeletal system drugs accounted for 9.0% of the reported ADRs (n = 121). Other notable categories included nervous system agents (n = 115, 8.6%), anti-infectives for systemic use (n = 114, 8.4%), and drugs affecting the blood and blood-forming organs (n = 94, 7.0%). Furthermore, a smaller percentage of ADRs were associated with genitourinary system and sex hormones (n = 61, 4.5%), antiparasitic products, insecticides, and repellents (n = 17, 1.3%), and various other drug classes (n = 10, 0.7%).

**Table 2.** Percentages of the top ACT drug classes associated with the reported ADRs.

ACT class	n (%)
Antineoplastic and immunomodulating agents	32 (2.3)
Anti-infectives for systemic use	114 (8.4)
Alimentary tract and metabolism	218 (16.2)
Nervous system	115 (8.6)
Musculo-skeletal system	121 (9.0)
Blood and blood forming organs	94 (7.0)
Cardiovascular system	221 (16.4)
Sensory organs	64 (4.6)
Dermatologicals	98 (7.3)
Systemic hormonal preparations, excluding sex hormones and insulins	110 (8.2)
Respiratory system	75 (5.5)
Various	10 (0.7)
Genitourinary system and sex hormones	61 (4.5)
Antiparasitic products, insecticides and repellents	17 (1.3)

Interestingly, among all reported drugs, 99.9% were considered suspected and 0.1% of the reported drugs were concomitant to ADRs. The route to administration of the reported drug are summarized in Table 3. The predominant route of administration among the reported ADRs was oral (n = 1199, 89.5%), indicating that the majority of adverse reactions occurred through this route. Intravenous administration accounted for a smaller percentage (n = 60, 4.5%) of the reported ADRs. Subcutaneous (n = 22, 1.8%) and intramuscular (n = 11, 0.7%) routes were less common in comparison.

**Table 3.** Characteristics of drugs involved in ADRs (n = 1340)

Characteristic	n (%)
**Drug role**	
Suspect/interacting	1339 (99.9)
Concomitant	1 (0.7)
**Route of administration**	
Oral	1199 (89.5)
Intravenous	60 (4.5)
Subcutaneous	22 (1.8)
Intramuscular	11 (0.7)
Topical	14 (0.8)
Ocular	7 (0.5)
Inhaled	27 (2.2)

## 4. Discussion

The current study provides a contemporary overview of the ADR reporting trend within a teaching hospital spanning a 7-month period. It reveals an average monthly ADR report count of 191, significantly surpassing previous findings from a pharmacovigilance database analysis in Jordan (85 per year) between 2010 and 2014 (Alsbou et al., 2017) and (108 per 11 months) in a Jordanian teaching hospital (ALsbou et al., 2015). This surge in ADR reporting signifies heightened awareness and understanding among healthcare providers regarding pharmacovigilance. It underscores the effective collaboration between pharmacists at a local pharmacovigilance center and regulatory bodies like the JFDA, streamlining the reporting process and organising various educational workshops and campaigns on pharmacovigilance.

In general, ADR reporting remains below optimal levels and requires further improvement by healthcare professionals. Under-reporting of ADRs is widespread internationally, as most countries utilise a voluntary and spontaneous reporting system. Consistent with previous research, our study found that ADR reports were more frequent among females and predominantly observed in adult patients (Shroukh et al., 2018; Yousef et al., 2022).

The three primary drug classes associated with reported ADRs, in descending order, were cardiovascular system (16.4%), alimentary tract and metabolism (16.2%), and musculoskeletal system (9.0%). Anti-infective agents, followed by blood and blood-forming agents, and musculoskeletal system medications were the most commonly reported ATC classes. These findings were consistent with previous studies conducted in Jordan (Alsbou et al., 2017), Ethiopia (Thakare et al., 2022), and Saudi Arabia (Yousef et al., 2022). However, studies from other countries like Germany have reported nervous system agents as the primary contributors to ADR reporting or hospitalisation (Li et al., 2021; Thomas et al., 2022). In our study, ADRs related to the nervous system accounted for 8.6% of the total. The high prevalence of these ATC classes in ADRs may be attributed to their frequent prescription for various prevalent diseases, misuse, particularly with anti-infective agents, or inappropriate use of other medications. Nonetheless, these findings should be interpreted cautiously as not all reported ADRs have a clear causality assessment with the suspected drug.

Abdominal pain, nausea, headache, and dizziness were the top four reported ADRs, indicating they were likely of mild to moderate severity, as only 2.4% resulted in serious outcomes. These findings are consistent with a previous analysis in Jordan, which identified the skin and subcutaneous, gastrointestinal, and nervous systems as the most frequently affected (Alsbou et al., 2017). However, they differ from observations in Brazil (dos Santos & Coelho, 2006) and India (Tripathy et al., 2021), where skin and cutaneous tissue were primarily impacted by ADRs among children. Our data align with a global analysis of ADRs submitted to the WHO database, VigiBase, which identified general disorders and administration site conditions as the major systems affected by ADRs (Aagaard et al., 2012).

Pharmacists played a significant role in ADR reporting, which is not surprising given their expertise in drug information. Previous studies have shown that pharmacist-led interventions can reduce medication and prescribing errors and improve the quality of ADR reporting. Therefore, efforts should be made to enhance pharmacists’ skills in detecting, monitoring, and reporting ADRs in Jordan.

The study provides a comprehensive analysis of ADR reporting patterns in a teaching hospital, contributing to post-marketing surveillance of medications. However, its retrospective nature limits conclusions about causal associations. The authors acknowledge the limitation that the present study is based solely on ADR reports collected from February to August 2023 at a single tertiary teaching hospital in Jordan. This restricted timeframe and geographical scope may limit the generalizability of our findings beyond this specific context. Additionally, caution should be exercised when generalising these findings to broader populations or other regions. Variations in healthcare practices, patient demographics, prescribing behaviours, and reporting systems across different settings could potentially influence the incidence and characteristics of ADRs observed. The present study relies on voluntary ADR reporting, which may lead to under-reporting or selective reporting bias. This potential bias could affect the comprehensiveness and accuracy of the data analysed. Despite these limitations, this study has employed robust methodologies to analyse and interpret the available data, including statistical measures to account for potential biases. Furthermore, it offers valuable insights into drug safety and ADR reporting patterns that may guide future prospective or interventional pharmacovigilance investigations.

## 5. Conclusions

Based on national data, there was a significant increase in spontaneous adverse drug reaction (ADR) reporting within a teaching hospital setting during the study period, highlighting the critical need for enhanced pharmacovigilance practices. This underscores the importance of continuous improvement in ADR tracking and reporting systems, especially among pharmacists and other healthcare providers. Future investigations should delve into the underlying factors and mechanisms driving the occurrence of prevalent serious ADRs. Such research will facilitate the implementation of corrective clinical and regulatory measures aimed at enhancing patient safety and health outcomes.

## Ethics approval and consent to participate

Ethical approval to conduct the study was obtained from the Institutional Review Board Committee at Jordan University Hospital (10/2022/8379).

## Availability of data and materials

The data that support the findings of this study are available from the corresponding author upon reasonable request.
